# Stroop interference in a delayed match-to-sample task: evidence for semantic competition

**DOI:** 10.3389/fpsyg.2013.00842

**Published:** 2013-11-15

**Authors:** Bradley R. Sturz, Marshall L. Green, Lawrence Locker, Ty W. Boyer

**Affiliations:** Department of Psychology, Georgia Southern UniversityStatesboro, GA, USA

**Keywords:** stroop, interference, delayed match-to-sample, semantic competition, response competition

## Abstract

Discussions of the source of the Stroop interference effect continue to pervade the literature. *Semantic competition* posits that interference results from competing semantic activation of word and color dimensions of the stimulus prior to response selection. *Response competition* posits that interference results from competing responses for articulating the word dimension vs. the color dimension at the time of response selection. We embedded Stroop stimuli into a delayed match-to-sample (DMTS) task in an attempt to test semantic and response competition accounts of the interference effect. Participants viewed a sample color word in black or colored fonts that were congruent or incongruent with respect to the color word itself. After a 5 s delay, participants were presented with two targets (i.e., a match and a foil) and were instructed to select the correct match. We probed each dimension independently during target presentations via color targets (i.e., two colors) or word targets (i.e., two words) and manipulated whether the semantic content of the foil was related to the semantic content of the irrelevant sample dimension (e.g., word sample “red” in blue font with the word “red” as the match and the word “blue” as the foil). We provide evidence for Stroop interference such that response times (RTs) increased for incongruent trials even in the presence of a response option with semantic content unrelated to the semantic content of the irrelevant sample dimension. Accuracy also deteriorated during the related foil trials. A follow-up experiment with a 10 s delay between sample and targets replicated the results. Results appear to provide converging evidence for Stroop interference in a DMTS task in a manner that is consistent with an explanation based upon semantic competition and inconsistent with an explanation based upon response competition.

## Introduction

Stroop interference is the well-known increased response time (RT) for naming font colors of incongruent color words (e.g., “red” in blue font) compared to font colors of congruent color words (i.e., “red” in red font; Stroop, 1935). The importance of such interference effects cannot be overstated, as they have been critical in the development of prominent theories of attention, cognitive processing, and executive function (e.g., Cohen et al., 1990; see also MacLeod, 1991, 1992) and considered diagnostic in clinical applications (Dalgleish, 1990; Nigg et al., 2005; see also Dimoska-Di Marco et al., 2011). Despite the widespread use of Stroop tasks, much theoretical and empirical debate remains concerning the source of interference (Luo, 1999; Stolz and Besner, 1999; Augustinova et al., 2010; see also, MacLeod, 1991, 1992).

Some suggest that an incongruent Stroop stimulus activates semantic representations in both the word and the font color dimensions, resulting in *semantic competition* between dimensions prior to response selection (Luo, 1999; Catena et al., 2002). Specifically, color words and colors both activate a semantic representation, and competition between these representations during incongruent trials requires active suppression of one representation which slows responding. Such competition is absent during congruent trials because the word and font color activate a representation from the same dimension. By contrast, *response competition* suggests that Stroop interference results from competing responses for *articulating* the word dimension vs. the color dimension at the time of response selection because both the color word and the font color activate a response (Besner et al., 1997; Stolz and Besner, 1999; for a review, see MacLeod, 1991, 1992). Competition between responses for each stimulus dimension during incongruent trials requires active suppression of a response from one dimension, which slows responding. Such response competition is absent during congruent trials because the response is identical for both dimensions.

Innovative tasks have separated font color and word dimensions using stimulus onset asynchrony manipulations and have utilized manual responses to eliminate articulation effects (Glaser and Glaser, 1982; Luo, 1999; De Houwer, 2003; Schmidt and Cheesman, 2005). Neural imagining techniques have also been utilized to delineate associated brain regions (Pardo et al., 1990; Taylor et al., 1997; Leung et al., 2000; for a review, see MacLeod and MacDonald, 2000). Yet, despite these advances, theoretical debate regarding the source of interference appears to remain a contested topic in contemporary literature (e.g., Luo, 1999; Stolz and Besner, 1999; Augustinova et al., 2010; see also, MacLeod, 1991, 1992). As a result, developing alternative tasks to investigate semantic and response competition appear ideal for providing converging evidence, clarifying existing theoretical accounts, and illuminating the extent to which such interference effects are observed in novel tasks incorporating Stroop stimuli.

Historically, Stroop tasks have analyzed RT as the critical measure of performance (for a review, see MacLeod, 1991, 1992). Accuracy measures, by contrast, have largely been neglected in this line of research; however, if embedded within an appropriately designed task, accuracy measures may corroborate RT measures regarding the cognitive processing involved in Stroop interference and assist in dissociating semantic and response competition. To that end, we embedded Stroop stimuli into a delayed match-to-sample (DMTS) task to investigate potential interference effects on both RTs and accuracy. In a DMTS task (see Wright, 2006), a trial begins with presentation of a sample stimulus for a predetermined duration, after which it is removed. Following a predetermined retention interval, two target stimuli are presented. One target is identical to the sample (i.e., a match) and the other is different (i.e., a foil). A response to the match is rewarded. Each trial contains a correct response, and accuracy provides critical information regarding the fidelity and duration of memory (Wright, 1992, 1997; Bodily et al., 2008; see also Wright, 2006).

**Figure 1:**
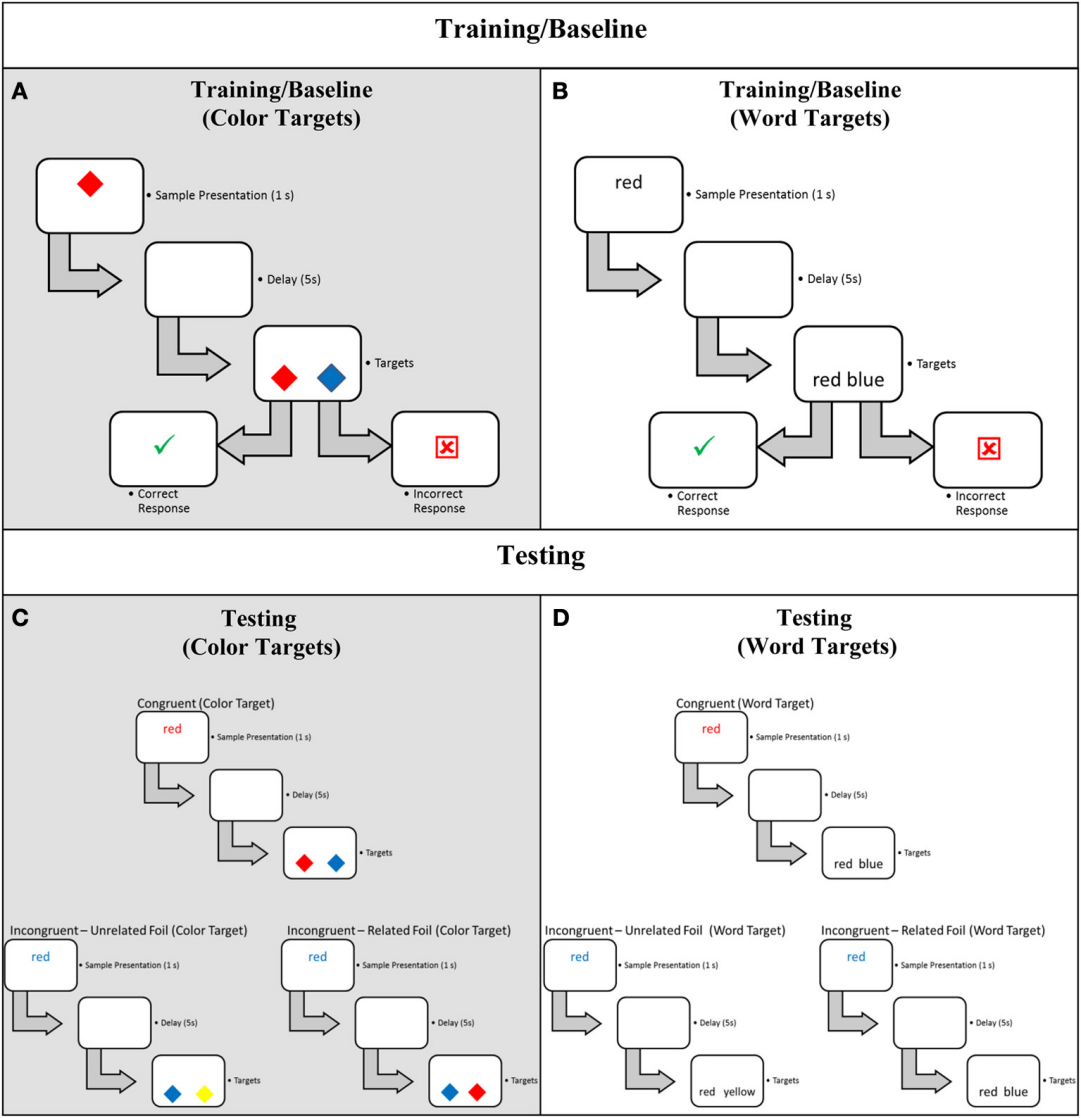
**Sample Trial Types and trial structures for the Delayed Match-to-Sample task.** One sample Baseline/Training trial is illustrated for Color Targets **(A)** and Word Targets **(B)**, and one sample Congruent, Incongruent—Unrelated Foil, and Incongruent—Related Foil trial is illustrated for Color Targets **(C)** and Word Targets **(D)**. For illustrative purposes, all correct matches are shown as the left target even though correct target and foil target locations were balanced (see text for details).

Unlike a standard DMTS task, we presented Stroop word samples that contained two dimensions; sample font color reflected its color dimension and sample word reflected its word dimension. We presented word samples in either black, congruent (e.g., “red” in red), or incongruent (e.g., “red” in blue) font colors. After a delay, we probed each dimension independently during target presentation via color targets (i.e., two colors) or word targets (i.e., two words; see Figure 1, panels **A,C** and **B,D**, respectively). Participants were instructed to select the correct match (i.e., select sample color if color targets; select sample word if word targets; see Figure 1 panels **A,C** and **B,D**, respectively). Thus, participants needed to attend to and potentially recall both color and word sample dimensions on every trial to respond accurately because target dimension cued the relevant to-be-recalled sample dimension.

We adopted the DMTS task for several reasons. First, this framework provides an intriguing opportunity to manipulate the congruency of the sample font color with respect to the sample color word. To be specific, a sample word could be presented in a neutral font color (e.g., black), a congruent font color (e.g., “red” in red), or an incongruent font color (e.g., “red” in blue). Second, this framework also enables manipulation of the extent to which the semantic content of the foil is related to the semantic content of the irrelevant sample dimension. To be specific, a foil could be presented that does not contain semantic content related to either the semantic content of the sample font color or the semantic content of the sample word (i.e., unrelated foil). For example, if the sample font color were blue and the sample word were “red,” two color targets could be presented in which the color blue could be the correct match but the color yellow could be the foil (or if two word targets were presented the correct match could be the word “red” but the foil could be the word “yellow”). Alternatively, a foil could be presented that does contain semantic content related to the semantic content of the irrelevant sample dimension (i.e., related foil). For example, if the sample font color were blue and the sample word were “red,” two color targets could be presented in which the color blue could be the correct match and the color red could be the foil (or if two word targets were presented the correct match could be the word “red” but the foil could be the word “blue”). As a result, this combination of manipulations in the DMTS task provides an opportunity to investigate the influence of congruency of the sample font color with respect to the sample color word (i.e., congruent vs. incongruent sample) as well as the extent to which the semantic content of the foil is related to the semantic content of the irrelevant sample dimension (i.e., unrelated vs. related foil). Third, as noted above, the DMTS task provides an opportunity to examine both accuracy and RT aspects of performance to assist in dissociating semantic and response competition.

The adopted DMTS task is ideal for testing semantic and response competition because manipulation of the congruency of the sample provides an opportunity to investigate semantic competition whereas the manipulation of the semantic relatedness of the foil provides an opportunity to investigate response competition. As a result, the DMTS allows an investigation of whether semantic competition only, response competition only, or both semantic and response competition influence performance. Specifically, determination of an appropriate response could result from *suppression of the irrelevant semantic content of the sample* (i.e., semantic competition), *suppression of the irrelevant response option* (i.e., response competition), or the *suppression of both the semantic content of the irrelevant sample dimension and the suppression of the irrelevant response option*.

Under the prevailing assumption that suppression slows responding, if suppression of the semantic content of the irrelevant *sample dimension* is involved in determining the appropriate response, RTs on trials in which the color word is presented in black (i.e., Baseline trials, see Figures 1A,B) or congruent font colors (i.e., Congruent trials, see Figures 1C,D) should not differ because these trials do not require the suppression of the semantic content of the irrelevant sample dimension (i.e., only one sample dimension is present on these trials) to determine the appropriate response. Importantly, Baseline and Congruent trials should be faster than trials in which the color word is presented in an incongruent font color (i.e., Incongruent—Unrelated Foil and Incongruent—Related Foil trials, see Figures 1C,D) because these incongruent trials would require the suppression of the semantic content of the irrelevant sample dimension to determine the appropriate response. Alternatively, if suppression of the semantic content of the irrelevant *response option* is involved in determination of the appropriate response, RTs on Baseline, Congruent, and Incongruent—Unrelated Foil trials should not differ because these trials do not require suppression of a response option with semantic content related to the irrelevant sample dimension (i.e., the semantic content of the foil is unrelated to the semantic content of either sample dimension). Importantly, Baseline, Congruent, and Incongruent—Unrelated Foil trials should be faster than Incongruent—Related Foil trials because Incongruent—Related Foil trials would require the suppression of a response option with semantic content related to the semantic content of the irrelevant sample dimension. Finally, if suppression of the semantic content of the irrelevant *sample dimension* and suppression of the semantic content of the irrelevant *response option* are involved in the determination of the appropriate response, then RTs for trials in which the color word is presented in black or congruent font colors should not differ, but these trials should be faster than Incongruent—Unrelated Foil trials. Importantly, Incongruent—Unrelated Foil trials should be faster than Incongruent—Related Foil trials because, under additive logic, RTs for Baseline and Congruent trials would require no suppression, Incongruent—Unrelated Foil trials would require suppression of the semantic content of the irrelevant sample dimension, and Incongruent—Related Foil trials would require the suppression of the semantic content of the irrelevant *sample dimension* and the suppression of a *response option* with semantic content related to the irrelevant sample dimension.

## Experiment 1

To explicitly test the prediction outlined above, we embedded Stroop stimuli into a DMTS task. Participants viewed a color word in black or colored fonts that were congruent or incongruent with respect to the color word itself. After a 5 s delay, participants were presented with two targets (i.e., a match and a foil) and were instructed to select the correct match. We probed each dimension independently during target presentations via color targets (i.e., two colors) or word targets (i.e., two words). We manipulated the congruency of the sample (i.e., congruent vs. incongruent sample) and whether the semantic content of the foil was related to the semantic content of the irrelevant sample dimension (i.e., unrelated vs. related foil).

### Method

#### Participants

Twenty undergraduate students at Georgia Southern University (6 males; 14 females) served as participants. Participants had normal or corrected-to-normal vision and received extra class credit or participated as part of a course requirement.

#### Apparatus

We constructed and implemented a DMTS task (see Figure 1) on a personal computer with a 22-inch flat-screen liquid crystal display (LCD) monitor (1680 × 1050 pixels). Responses occurred via the “c” (left target) and “m” (right target) keys on a standard keyboard. Experimental events were controlled and recorded using E-Prime (Psychology Software Tools, Inc., www.pstnet.com).

#### Stimuli

There were two stimulus types: Colors and Words. Color stimuli were blue, red, and yellow patches presented as a 410 × 410 pixel filled diamond subtending 9.6^°^ visual angle horizontally and vertically (Figures 1A,C). Word stimuli were “blue,” “red,” and “yellow” (Figures 1B,D) presented in bold 48 point Courier New font and were 112 (“red”), 149 (“blue”), and 228 (“yellow”) pixels in width, subtending 2.6^°^ (“red”), 3.5^°^ (“blue”), and 5.4^°^ (“yellow”) visual angle horizontally, and 40 (“red,” “blue”) or 52 (“yellow”) pixels in height, subtending 0.9 or 1.2^°^ visual angle vertically. Depending on trial type (see below and Figure 1), words were presented in black, blue, red, or yellow font color. All stimuli were presented on a white background. Samples were presented in the horizontal center of the screen 25% down from its top edge. Targets were presented on opposite sides of the screen, 50% of screen width apart, and 25% up from its bottom edge.

#### Procedure

We provided participants with an instruction page that informed them that they would complete a memory test in which one of several words would appear on the screen in one of several colors and that either a pair of words or a pair of shapes would then appear. Instructions told them that their task would be to select the word that matched the first word (if word pairs) or select the shape that matched the color of the first word (if shape pairs).

The experimental protocol consisted of 120 total trials for each participant. The 120 total trials consisted of 24 Training Trials and 96 Testing Trials (see below). On each trial, we presented samples for 1 s, followed by a 5 s blank screen retention interval delay, followed by target stimuli for 1.5 s. We provided participants with response feedback. A response to the correct target (i.e., match) resulted in the presentation of a green check mark; a response to the incorrect target (i.e., foil) resulted in the presentation of a red “X,” and failure to respond during the 1.5 s target presentation produced a “No Response” statement. Feedback was presented for 1 s, and served as the inter-trial interval (ITI).

***Training.*** To familiarize participants with the task and train them to match a color sample to a color target and a word sample to a word target, we provided them with 24 training trials composed of two 12-trial blocks. One block included 12 unique color training trials in which participants matched a sample color to its corresponding color target (Figure 1A), and the other block included 12 unique word training trials in which participants matched a sample word to its corresponding word target (Figure 1B). Word Samples and Word Targets were presented in black font for the duration of Training. We counterbalanced the training blocks order of presentation.

***Testing.*** Testing consisted of 96 trials composed of 12 eight-trial blocks. Each trial block was composed of two trials of each of four trial types: Baseline (Training), Congruent (sample word in corresponding font color), Incongruent—Unrelated Foil (sample word in non-corresponding font color, but the semantic content of a foil unrelated to the semantic content of the irrelevant sample dimension), and Incongruent—Related Foil (sample word in non-corresponding font color, but the semantic content of a foil related to the semantic content of the irrelevant sample dimension). Figure 1 illustrates all trial types. Baseline trials were identical to Training trials.

For Congruent, Incongruent—Unrelated Foil, and Incongruent—Related Foil trials, when word targets were presented (e.g., “red” and “blue”), they were presented in black font throughout Testing, and the corresponding sample word was the correct response. When color targets were presented (e.g., red and blue), the corresponding sample font color was the correct response. Congruent trials presented the sample word in its corresponding font color (e.g., “red” in red font). Incongruent—Unrelated Foil trials presented the sample word in a non-corresponding font color (e.g., “red” in blue font) but the semantic content of the foil was unrelated to the semantic content of the irrelevant sample dimension (i.e., word targets of “red” and “yellow”; color targets of red and yellow). Incongruent—Related Foil trials also presented the sample word in a non-corresponding font color (e.g., “red” in blue font); however, the semantic content of the foil was related to the semantic content of the irrelevant sample dimension (i.e., word targets of “red” and “blue”; color targets of red and blue).

For all trial types within each block, we presented one trial with color targets and one trial with word targets. The trial type sequence was randomized within each block. The left/right location of the correct target (i.e., match) and the foil was counterbalanced, which resulted in each unique combination of each trial type being presented once, without replacement, for a total of 96 trials during Testing (24 Baseline trials, 24 Congruent trials, 24 Incongruent—Unrelated Foil trials, and 24 Incongruent—Related Foil trials). Feedback was identical to Training.

### Results

We analyzed Testing data via RTs and proportions correct.

#### Response time

**Figure 2:**
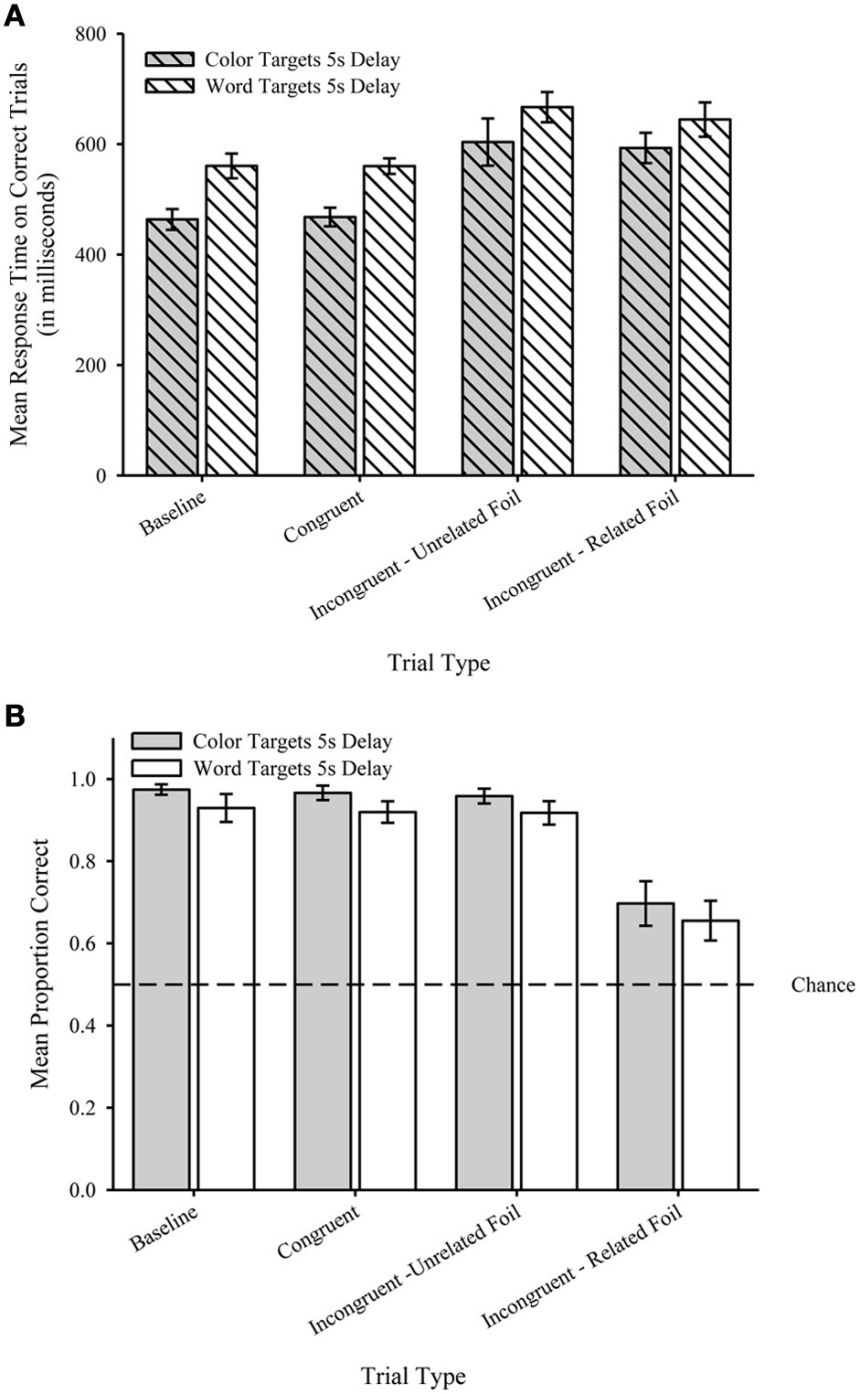
**(A)** Mean response time during Testing (in milliseconds) plotted by Trial Type for Color Targets (filled bars) the Word Targets (unfilled bars) for Experiment 1. **(B)** Mean proportion correct during Testing plotted by Trial Type for Color Targets (filled bars) the Word Targets (unfilled bars) for Experiment 1. Dashed line represents chance performance (0.5). Error bars represent standard errors of the means.

We analyzed correct trials (error rates opposite of proportion correct shown Figure 2B). Overall, participants were faster to respond to Color Targets (*M* = 511.7, *SEM* = 16.4) compared to Word Targets (*M* = 595.8, *SEM* = 15.6). Figure 2A shows the mean RTs (in ms) plotted by Trial Type for Color Targets (filled) and Word Targets (unfilled)^1^. A two-way repeated measures analysis of variance (ANOVA) with Target Type (color targets, word targets) and Trial Type (baseline, congruent, incongruent—unrelated foil, incongruent—related foil) as the within-subject factors revealed a main effect of Target Type, *F*_(1, 17)_ = 59.48, *p* < 0.001, η^2^_p_ = 0.78, and a main effect of Trial Type *F*_(3, 51)_ = 19.28, *p* < 0.001, η^2^_p_ = 0.53. The interaction was not significant, *F*_(3, 51)_ = 0.97, *p* = 0.42. *Post hoc* tests on the Trial Type factor revealed that Baseline (*M* = 494.6, *SEM* = 14.1) and Congruent (*M* = 500.1, *SEM* = 11.7) trials were significantly faster than both Incongruent—Unrelated Foil (*M* = 603.0, *SEM* = 22.0) and Incongruent—Related Foil (*M* = 617.3, *SEM* = 27.5) trials (*p*s < 0.001), but Baseline and Congruent trials were not significantly different from each other (*p* = 0.5). Importantly, Incongruent—Unrelated and Incongruent—Related Foil trials were not significantly different from each other (*p* = 0.49).

#### Proportion correct

We eliminated trials in which participants failed to respond ^2^—resulting in the elimination of 45 of the total 1920 trials (1.6% total). Overall, participants were more accurate with Color Targets (*M* = 0.89; *SEM* = 0.02) compared to Word Targets (*M* = 0.86; *SEM* = 0.02). Figure 2B shows the mean proportion correct plotted by Trial Type for Color Targets (filled) and Word Targets (unfilled). A two-way repeated measure ANOVA on proportion correct with Target Type (color targets, word targets) and Trial Type (baseline, congruent, incongruent—unrelated foil, incongruent—related foil) as the within-subject factors revealed a main effect of Target Type, *F*_(1, 19)_ = 4.76, *p* < 0.05, η^2^_p_ = 0.2, and a main effect of Trial Type, *F*_(3, 57)_ = 55.5, *p* < 0.001, η^2^_p_ = 0.75. The interaction was not significant, *F*_(3, 57)_ = 0.0, *p* = 1.0. *Post hoc* tests on the Trial Type factor revealed that Incongruent—Related Foil trials (*M* = 0.68, *SEM* = 0.03) were significantly less accurate than Baseline (*M* = 0.95, *SEM* = 0.02), Congruent (*M* = 0.94, *SEM* = 0.02), and Incongruent—Unrelated Foil (*M* = 0.94, *SEM* = 0.02) trials (*p*s < 0.001), but Baseline, Congruent, and Incongruent—Unrelated Foil trials were not significantly different from each other (*p*s > 0.34). One-sample *t*-tests revealed that mean proportion correct was significantly greater than chance (0.5) for all Trial Types and Target Types, *t*s_(19)_ > 3.2, *p*s < 0.01.

### Discussion

The RT analyses indicated that responses for both incongruent trial types took longer than both Baseline and Congruent trials, but Baseline and Congruent trials were not significantly different from each other. Importantly, the Incongruent—Unrelated Foil and Incongruent—Related Foil trials were not significantly different from each other. The accuracy analyses indicated a decrement in performance for incongruent trials in which the semantic content of the foil was related to the irrelevant sample dimension. Both the RT and accuracy measures indicated superior performance for color targets compared to word targets.

RTs only increased on trials in which the sample was presented in an incongruent font color. Given that Baseline and Congruent trials removed the requirement to suppress the semantic content of the irrelevant sample dimension, we attribute this RT increase on these incongruent trial types to the suppression of the semantic content of the irrelevant sample dimension. Because Baseline, Congruent, and Incongruent—Unrelated Foil trials removed the requirement to suppress the semantic content of a response option related to the semantic content of the irrelevant sample dimension, the increase in RT for the Incongruent—Unrelated Foil trials cannot be attributed to response competition. As importantly, the non-significant difference in RTs for Incongruent—Unrelated Foil and Incongruent—Related Foil trials suggest that only semantic competition influenced performance because had both semantic and response competition influenced performance, we would have expected longer RTs for Incongruent—Related Foil compared to Incongruent—Unrelated Foil trials. As a result, we interpret these results as consistent with a semantic competition (e.g., Luo, 1999; Catena et al., 2002) but inconsistent with a response competition (e.g., Besner et al., 1997; Stolz and Besner, 1999) account of Stroop interference. Such an interpretation may also explain the superior performance for color targets compared to word targets because of the potential for increased difficulty in suppressing the semantic content of a word dimension compared to the semantic content of a color dimension.

Despite the increased RTs for both incongruent trial types, accuracy was only affected in the related foils condition. In short, our results appear to be opposite of a speed-accuracy trade-off and corroborate an interpretation consistent with semantic competition. Given the evidence for semantic competition reflected in the RTs, we attribute the relatively high accuracy rates in the Baseline and Congruent trials to the fact that these trials removed the requirement to suppress an irrelevant sample dimension to determine the appropriate response. Similarly, we attribute the relatively high accuracy rates in the Incongruent—Unrelated Foils trials to the fact that any difficulty in suppressing the semantic content of the irrelevant sample dimension would not disrupt an ability to determine the appropriate response. More specifically, on trials in which the semantic content of the foil is unrelated to the semantic content of the irrelevant sample dimension, (i.e., Incongruent—Unrelated Foil trials), any difficulty in suppressing the semantic content of the irrelevant sample dimension would not be expected to be reflected in accuracy because the semantic content of one target (i.e., the match) would match the semantic content of the relevant sample dimension and the semantic content of the other target (i.e., the foil) would not match the semantic content from the irrelevant sample dimension. In contrast, we attribute the relatively low accuracy on the Incongruent—Related Foils trials to the fact that any difficulty in suppressing the semantic content of the irrelevant sample dimension would disrupt an ability to determine the appropriate response. More specifically, on trials in which the foil is related to the semantic content of the irrelevant sample dimension (i.e., Incongruent—Related Foil trials), any difficulty in suppressing the semantic content of the irrelevant sample dimension would be expected to be reflected in accuracy because the semantic content of one target (i.e., the match) matches the semantic content of the relevant sample dimension and the semantic content of the other target (i.e., the foil) matches the semantic content of the irrelevant sample dimension. As a result, there is greater probability of error under the Incongruent—Related Foil trials due to two potential matches on the basis of semantic content.

Although a delay between sample and target presentations was a component of the DMTS task, the results cannot be attributed to greater cognitive load for the Incongruent—Related Foil compared to the Incongruent—Unrelated Foil trials. Specifically, both trial types involved the encoding and retention of two sample dimensions and the suppression of the semantic content of the irrelevant sample dimension. The only difference between these trials was that any difficulty in suppressing the semantic content of the irrelevant sample dimension on Incongruent—Related Foil trials would result in a greater probability of error due to two potential matches on the basis of semantic content. Given that RTs were not significantly different on both incongruent trial types but accuracy only decreased during related foil trials, it seems unlikely that these effects were due to a source other than a potential difficulty in suppressing the semantic content of the irrelevant sample dimension. As a result, we attribute the decrement in performance on the related foil trials to semantic competition. Such an interpretation would also explain the relatively high accuracy for all trial types other than that of the Incongruent—Related Foil trials because the conditions of these other trial types did not require the suppression of the semantic content of the irrelevant sample dimension (i.e., Baseline and Congruent trials) or did not contain semantic content of a foil related to the semantic content of the irrelevant sample dimension (Incongruent—Unrelated Foil trials).

To investigate whether the results obtained in Experiment 1 were dependent on task parameters, we conducted a follow-up experiment in which we extended the delay between sample and target presentation (i.e., increased the retention interval). We again manipulated congruency of the sample word with respect to its font color (i.e., congruent vs. incongruent sample) and the extent to which foils contained semantic information related to the irrelevant sample dimension (i.e., unrelated vs. related foil). Importantly, if suppression of the semantic content of the irrelevant sample dimension is involved in the determination of an appropriate response (as suggested by Experiment 1), we should replicate the effect of Trial Type obtained in Experiment 1 for both RT and accuracy measures of performance.

## Experiment 2

### Participants

Twenty undergraduate students (10 males; 10 females) different from those who participated in Experiment 1 served as participants. Participants had normal or corrected-to-normal vision and received extra class credit or participated as part of a course requirement.

### Apparatus, stimuli, and procedure

The apparatus, stimuli, and procedure were identical to Experiment 1 with the exception that we increased the duration between sample presentation and target presentation (i.e., increased the retention interval) to 10 s. As with Experiment 1, the experimental protocol consisted of 120 total trials for each participant. The 120 total trials consisted of 24 Training Trials and 96 Testing Trials. Samples were presented for 1 s, followed by a 10 s blank screen retention interval. As with Experiment 1, we counterbalanced the training blocks order of presentation.

### Results

As with Experiment 1, we analyzed Testing data via RTs and proportions correct.

#### Response time

**Figure 3:**
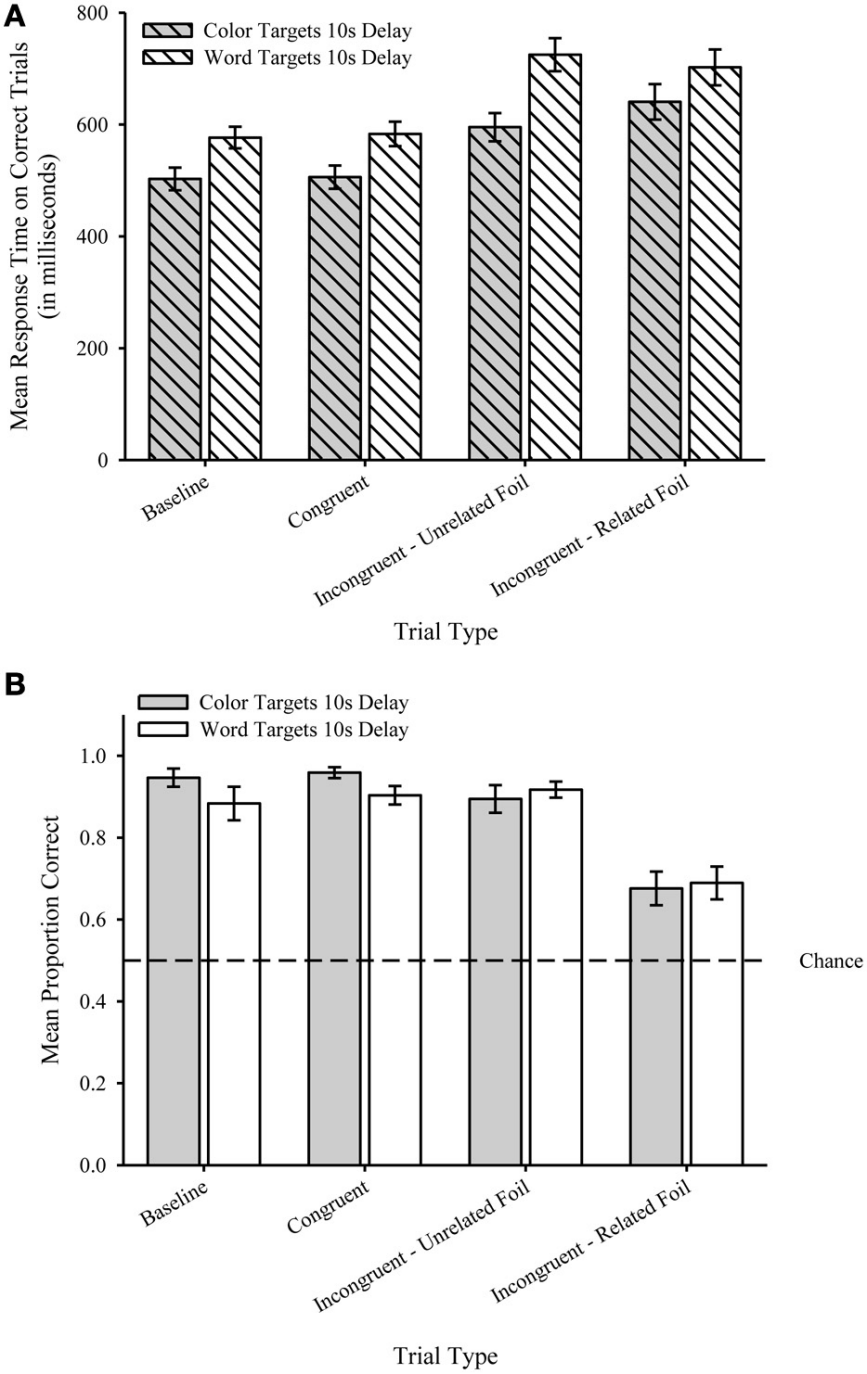
**(A)** Mean response time during Testing (in milliseconds) plotted by Trial Type for Color Targets (filled bars) the Word Targets (unfilled bars) for Experiment 2. **(B)** Mean proportion correct during Testing plotted by Trial Type for Color Targets (filled bars) the Word Targets (unfilled bars) for Experiment 2. Dashed line represents chance performance (0.5). Error bars represent standard errors of the means.

We analyzed correct trials (error rates opposite of proportion correct shown Figure 3B). As with Experiment 1, participants were faster to respond to Color Targets (*M* = 561.1, *SEM* = 19.9) compared to Word Targets (*M* = 646.8, *SEM* = 22.1). Figure 3A shows the mean RTs (in ms) plotted by Trial Type for Color Targets (filled) and Word Targets (unfilled). A two-way repeated measures ANOVA with Target Type (color targets, word targets) and Trial Type (baseline, congruent, incongruent—unrelated foil, incongruent—related foil) as the within-subject factors revealed a main effect of Target Type, *F*_(1, 19)_ = 54.23, *p* < 0.001, η^2^_p_ = 0.74, and a main effect of Trial Type *F*_(3, 57)_ = 31.1, *p* < 0.001, η^2^_p_ = 0.62. The interaction was not significant, *F*_(3, 57)_ = 1.93, *p* = 0.14. *Post hoc* tests on the Trial Type factor revealed that Baseline (*M* = 539.7, *SEM* = 18.2) and Congruent (*M* = 544.5, *SEM* = 18.8) trials were significantly faster than both Incongruent—Unrelated Foil (*M* = 660.1, *SEM* = 25.0) and Incongruent—Related Foil (*M* = 671.4, *SEM* = 28.6) trials (*p*s < 0.001), but Baseline and Congruent trials were not significantly different from each other (*p* = 0.4). Importantly, Incongruent—Unrelated and Incongruent—Related Foil trials were not significantly different from each other (*p* = 0.55).

#### Proportion correct

As with Experiment 1, we eliminated trials in which participants failed to respond ^3^—resulting in the elimination of 170 of the total 1920 trials (8.9% total). Participants were equally accurate with Color Targets (*M* = 0.87; *SEM* = 0.02) and Word Targets (*M* = 0.85; *SEM* = 0.03). Figure 3B shows the mean proportion correct plotted by Trial Type for Color Targets (filled) and Word Targets (unfilled). A two-way repeated measure ANOVA on proportion correct with Target Type (color targets, word targets) and Trial Type (baseline, congruent, incongruent—unrelated foil, incongruent—related foil) as the within-subject factors revealed only a main effect of Trial Type, *F*_(3, 57)_ = 39.4, *p* < 0.001, η^2^_p_ =0.68. Neither the effect of Target Type, *F*_(1, 19)_ = 1.75, *p* = 0.2, nor the interaction was significant, *F*_(3, 57)_ = 1.86, *p* = 0.15. *Post hoc* tests on the Trial Type factor revealed that Incongruent—Related Foil trials (*M* = 0.68, *SEM* = 0.03) were significantly less accurate than Baseline (*M* = 0.92, *SEM* = 0.03), Congruent (*M* =0.93, *SEM* = 0.02), and Incongruent—Unrelated Foil (*M* = 0.91, *SEM* = 0.02) trials (*p*s < 0.001), but Baseline, Congruent, and Incongruent—Unrelated Foil trials were not significantly different from each other (*p*s > 0.15). One-sample *t*-tests revealed that mean proportion correct was significantly greater than chance (0.5) for all Trial Types and Target Types, *t*s_(19)_ > 4.2, *p*s < 0.001^4^.

### Discussion

Results from Experiment 2 replicated the critical effects obtained in Experiment 1 such that RTs for both incongruent trial types took longer than both Baseline and Congruent trials, but Baseline and Congruent trials were not significantly different from each other. As importantly, RTs on Incongruent—Unrelated Foil and Incongruent—Related Foil trials were not significantly different from each other. As with Experiment 1, the accuracy analyses indicated a decrement in performance for incongruent trials in which the semantic content of the foil was related to the semantic content of the irrelevant sample dimension. Most importantly, Experiment 2 provides converging evidence that the pattern of results with respect to both RTs and accuracy measures can be attributed to involvement of suppression of the semantic content of the irrelevant sample dimension and not the suppression of the irrelevant response option. As a result, Experiment 2 provides converging evidence consistent with an interpretation based upon a semantic competition (e.g., Luo, 1999; Catena et al., 2002) but inconsistent with an interpretation based upon a response competition (e.g., Besner et al., 1997; Stolz and Besner, 1999) account of the Stroop interference effect.

## General discussion

Results from both DMTS experiments indicated that RTs for both incongruent trial types took longer than both Baseline and Congruent trial types and Baseline and Congruent trials were not significantly different from each other. As importantly, the RTs for the Incongruent—Unrelated Foil and Incongruent—Related Foil trials were not significantly different from each other. Both experiments also indicated relatively lower accuracy for incongruent trials in which the semantic content of the foil was related to the semantic content of the irrelevant sample dimension.

Collectively, we interpret these results as providing converging evidence that incongruent trial types involved the suppression of the semantic content of the irrelevant sample dimension in order to determine the appropriate response. Given that RTs increased on trials in which the sample was presented in an incongruent font color and that there was no significant difference between RTs on trials in which the foil was related or unrelated to the irrelevant sample dimension, we attribute the pattern of data to semantic competition. Because Baseline, Congruent, and Incongruent—Unrelated Foil trials removed the requirement to suppress a response option with semantic content related to the semantic content of the irrelevant sample dimension, the increase in RT for the Incongruent—Unrelated Foil trials relative to Baseline and Congruent trials cannot be attributed to response competition. As a result, we interpret these results as consistent with a semantic competition account (e.g., Luo, 1999; Catena et al., 2002) but inconsistent with a response competition account (e.g., Besner et al., 1997; Stolz and Besner, 1999) of Stroop interference.

We acknowledge that both semantic and response competition accounts appear to be silent with respect to error rates, but our inclusion of accuracy measures appears to corroborate the RT evidence for semantic competition, and both RT and accuracy measures provide evidence for interference effects under novel Stroop conditions. Regardless of potential disagreements about our characterization, interpretation, and application of these theoretical accounts of the standard Stroop interference effect to the adopted DMTS task, our results suggest that such interference effects can be observed in a novel task incorporating Stroop conditions and accounted for in a manner consistent with semantic competition (Luo, 1999; Catena et al., 2002; De Houwer, 2003; Schmidt and Cheesman, 2005). As a result, it remains unclear how a response competition account of the Stroop interference effect (e.g., Besner et al., 1997; Stolz and Besner, 1999) would explain the results from the present set of experiments. Specifically, it is unclear how such an account could explain the significant increase but non-significant difference on RTs for both Incongruent—Unrelated Foil and Incongruent—Related Foil trials even though Incongruent—Unrelated foil trials did not contain a response option with semantic content related to the semantic content of the irrelevant sample dimension.

Despite the fact that the present DMTS eliminated an articulatory response utilized in traditional Stroop tasks, it remains unclear how possible alternative forms of response competition may have influenced the present results. For example, bimanual or bidigit response interference was not eliminated and may have contributed to increased RTs and/or decreased accuracy. Future research may be able to utilize a touch screen apparatus such that potential bimanual or bidigit response interference could be minimized or incorporate concurrent vocal and behavioral responses to assist in further dissociating semantic and response competition (cf, Redding and Gerjets, 1977; Wijnen and Ridderinkhof, 2007; Proctor and Chen, 2012; Ferrand and Augustinova, 2013).

It may have been reasonable to suppose that semantic competition would be stronger during the Incongruent—Related Foils trials compared to the Incongruent—Unrelated Foils trials, but there was no statistical difference between these trials types with respect to RT. The decrement in accuracy, however, during the Incongruent—Related Foil trials may be reflective of the presence of this potentially greater semantic competition. Similarly, it seems reasonable to suppose that semantic facilitation would occur during Congruent trials (e.g., Dalrymple-Alford, 1972; Redding and Gerjets, 1977; for a review, see MacLeod, 1991, 1992), but there was no statistical difference between Baseline and Congruent trials in either measures of RT or accuracy. Consequently, the present results diverge slightly from previous research that has been able to obtain RT evidence for increased semantic competition and facilitation (e.g., Dalrymple-Alford, 1972; Redding and Gerjets, 1977; for a review, see MacLeod, 1991, 1992). Currently, the exact reasons for these differences between the present results and those that have obtained evidence for increased competition and facilitation remain unclear, but such differences may be a result of paradigmatic and/or parametric differences between the present task and the traditional Stroop task.

Despite these differences, we believe that the DMTS task adopted in the present experiments provides a novel approach to investigating semantic and response competition while incorporating measures of performance accuracy that may not be reflected in RTs. Importantly, the present DMTS task appears to capture the Stroop interference effect and appears to provide a novel paradigm in which to assess the activity/inactivity of specific brain areas related to semantic and response competition (cf, Van Veen and Carter, 2005; Kühn et al., 2011; Chen et al., 2013). Moreover, given the nature of the task, it seems ideal for phylogenic and ontogenic comparisons of Stroop-like interference effects. Such a task appears valuable for the study of various cognitive processes involved in Stroop performance such as attention, learning, information storage and retrieval, and decision making (see MacLeod, 2005; see also Wright, 2006).

Although interpretation of our RT and accuracy analyses appears consistent with a semantic competition account of the standard Stroop interference effect, we acknowledge the numerous differences between the present DMTS task and the traditional Stroop task. Even though we believe a strength of the current DMTS paradigm resides in its ability to capture robust interference effects with both RT and accuracy measures under novel task conditions, we reserve judgment about the extent to which the present results definitively implicate semantic competition as the source of Stroop interference in the traditional paradigm. Future research may be able to shorten the delay between sample and target presentations and more fully incorporate both reaction time and accuracy measures to closer approximate the traditional Stroop task for comparative purposes. By extension, we also reserve judgment about the extent to which the present results definitively eliminate a response competition account for the traditional Stroop effect (Besner et al., 1997; Stolz and Besner, 1999), but future research could continue to explore these interference effects utilizing our DMTS paradigm to illuminate these and related issues regarding semantic and response competition, the source of the interference effect, and the mechanisms underlying attention, cognitive processing, and executive function.

### Conflict of interest statement

The authors declare that the research was conducted in the absence of any commercial or financial relationships that could be construed as a potential conflict of interest.

## Footnotes

^1^One participant did not have any correct responses for Incongruent—Related Foil color targets and another participant did not have any correct responses for the Incongruent—Related Foil word targets. As a result, two cells were missing in the ANOVA which is reflected in the degrees of freedom.

^2^We also conducted identical analyses to those reported that included trials in which participants failed to respond (i.e., errors of omission), and the statistical results revealed only a main effect of Trial Type, *F*_(3, 57)_ = 48.99, *p* < 0.001, η^2^_p_ = 0.72. Neither the effect of Target Type nor the interaction was significant, *F*s < 1.4, *p*s > 0.25. Post hoc tests on the Trial Type factor revealed that Baseline trials did not significantly differ from Congruent trials (*p* = 0.51), but both were significantly more accurate than Incongruent—Unrelated Foils and Incongruent—Related Foil trials (*p*s < 0.05). In addition, Incongruent—Unrelated Foil trials were significantly more accurate than Incongruent—Related Foil trials (*p* < 0.01).

^3^We also conducted identical analyses to those reported that included trials in which participants failed to respond (i.e., errors of omission), and the statistical results revealed only a main effect of Trial Type, *F*_(3, 57)_ = 39.9, *p* < 0.001, η^2^_p_ = 0.68. Neither the effect of Target Type nor the interaction was significant, *F*s < 2.1, *p*s > 0.17. Post hoc tests on the Trial Type factor revealed that Baseline trials did not significantly differ from Congruent trials (*p* = 0.6), but both were significantly more accurate than Incongruent—Unrelated Foils and Incongruent—Related Foil trials (*p*s < 0.05). In addition, Incongruent—Unrelated Foil trials were significantly more accurate than Incongruent—Related Foil trials (*p* < 0.001).

^4^We also conducted across experiment analyses on both RTs and proportion correct as reported above including Experiment as a between-subjects factor. The main effect of Experiment was not significant, and Experiment did not interact with Target Type or Trial Type.
